# Investigating the Psychological Impact of Cyber-Sexual Harassment

**DOI:** 10.1177/08862605241231615

**Published:** 2024-02-15

**Authors:** Marvin Iroegbu, Freya O’Brien, Luna Clara Muñoz, Georgia Parsons

**Affiliations:** 1 Oxleas NHS Foundation Trust, Dartford, Kent, UK; 2 School of Justice Studies, Liverpool John Moores University, UK; 3 University of Liverpool, UK

**Keywords:** cyber-sexual, harassment, trauma, depression, anxiety, body image

## Abstract

The impact of cyber sexual harassment (CSH) on adult women and the factors influencing this impact are largely under-researched. Communication technologies provide novel means for people to threaten, communicate, and harass others. Victims of in-person sexual harassment (ISH) can experience negative symptoms of depression, anxiety, trauma, and negative body image. The current study explored the psychological impact of CSH in adult women to determine whether CSH predicts psychological difficulties. Adult female participants (*N* = 136) took part in an online, cross-sectional study; 44% of participants had experienced CSH and this was associated with higher levels of depression, anxiety, trauma, and body image dissatisfaction. Younger victims, those who had been in a relationship for a shorter amount of time, those who had previously experienced of ISH, and those who had a higher number of social media followers were more likely to have experienced CSH. When controlling for demographic variables, CSH predicted anxiety, depression, trauma, and body image dissatisfaction; however, experience of ISH impacted upon body-image dissatisfaction over and above CSH. There is a need to routinely ask individuals accessing mental health services whether online interactions cause harm. Future research should examine these phenomena in more ethnically diverse samples.

## Introduction

In the United Kingdom (UK), the Equality Act (2010) describes sexual harassment as occurring when a person engages “in unwanted sexual contact with another person.” This includes making inappropriate comments, jokes, or gestures and making unwanted physical contact with another person that ‘has the purpose or effect of either violating the other person’s dignity or creating an intimidating, hostile, degrading, humiliating or offensive environment for them.’ The issue of in-person sexual harassment (ISH) is extremely widespread, with figures suggesting 71% of women of all ages in the UK have been victimized (UN Women UK, 2021). Victims of ISH report higher levels of depression (Marsh et al., 2009), anxiety (Richman et al., 1999), trauma (e.g., Bendixen et al., 2018), negative body image (Fisher et al., 2019), eating disorder psychopathology (Hayes et al., 2021), and increased sexual risk-taking (Norcott et al., 2021) across several different settings. The growth in technological advancement and social media have contributed to new ways for women to be harassed (Craker & March, 2016). However, there is a lack of research examining adult victimization of sexual harassment online [cyber-sexual harassment, [CSH]) (Powell & Henry, 2018). CSH has been viewed as an extension of ISH (Li, 2005) with three related but conceptually distinct dimensions (Fitzgerald et al., 1995): (a) Verbal or graphic gender harassment (e.g., sending gender humiliating images); (b) Unwanted online sexual attention (e.g., sending unwanted sex-related messages); (c) Sexual coercion (e.g., pressuring an individual to perform sexual acts online). As the world becomes increasingly digitized, it is important to explore the impact of CSH on the women who experience it. If victimization causes negative psychological consequences, this knowledge can be used to inform clinical interventions and begin therapeutic conversations around online experiences (Stahl & Dennhag, 2021). Hence, the current study aims to examine the psychological impact of CSH on victims.

The integrated biopsychosocial (BPS) framework can explain how sexual harassment, in general, can lead to negative well-being (Knapp et al., 2023) and could explain possible ways CSH may lead to similar outcomes. Experiences of harassment can be negatively appraised as interpersonal stressors (e.g., Reddy & Murdoch, 2016) or threats (Lazarus & Folkman, 1984). Appraising the stressor with fear (e.g., of reprisal) can lead to victims feeling a loss of control or autonomy (Duncan et al., 2019), which may directly impact well-being (e.g., Greenwood et al., 2014). Individuals will respond to the stress with different types of coping strategies (e.g., Folkman, 2011), including avoidance and/or denial of the event (e.g., Bergman et al., 2002). Over time, these strategies could impact feelings of self-esteem, optimism, and personal control (Knapp et al., 2023). Individuals may feel worthless, rejected, negative, and less autonomous (Duncan et al., 2019) and be more likely to experience negative states of well-being, including anxiety, depression, and trauma (Knapp et al., 2023). When harassment is online, CSH may be felt more pervasively than ISH due to the permanence and speed at which online material is shared; threatening messages can be instantaneously communicated through online means and sent to an unlimited number of people unexpectedly and often anonymously (Durán & Rodriguez-Dominguez, 2023). Victims may feel as if it would be difficult to bring perpetrators to justice and may feel a lack of control and powerlessness over victimization (Scarduzio et al., 2018). Further, the speed and pervasiveness of this form of abuse may mean that victims’ ability to appraise threats effectively may be compromised (e.g., due to information processing biases; Hart, 2014). This may then cause significant distress and provoke anxiety (Straude-Muller et al., 2012).

CSH victimization may also impact upon victims’ feelings about their own body. The cognitive model of body image disturbance, individuals with negative body image are more likely to harshly judge their own appearance and interpret the behavior of others based on their own beliefs about their body (Lewis-Smith et al., 2019). If an individual responds to an incident of CSH by drawing on their pre-existing beliefs about their body as a method of coping, this could in turn lead to issues such as low self-esteem around appearance and have a negative psychological impact on victims. Similarly, objectification theory may also help to explain how CSH can impact on body image. According to objectification theory, repeated experiences of women being sexually objectified may lead to individuals habitually monitoring their body’s outward appearance, in what is described as body surveillance (Fredrickson & Roberts, 1997). The increased levels of body-monitoring may lead to greater levels of self-objectification. Sexual objectification has been associated with higher levels of self-objectification in women who had reported experiencing sexual violence from strangers (Fairchild & Rudman, 2008). Additionally, women who experience a higher frequency of CSH also display higher levels of self-objectification and eating pathology (Oliver et al., 2023). Therefore, women who are increasingly sexualized may self-objectify more, leading to greater dissatisfaction with their body image which leads to pathological eating habits to exert more control over their body’s appearance (Oliver et al., 2023).

The psychological impact of CSH victimization has mostly been investigated in children and adolescents (Powell et al., 2018) with findings suggesting negative mental health outcomes (e.g., Stahl & Dennhag, 2021). Studies examining victimization in adult women generally measure general cyber harassment which can include CSH (Ahmed et al., 2021), investigate negative impacts in predominately university student samples (Cripps & Stermac, 2018), examine broader psychological impacts such as feeling upset (Powell & Henry, 2019), consider specific behaviors within CSH (Durán & Rodriguez-Dominguez, 2023), and give insights into harm felt by victims from the perspectives of the police and practitioners (Powell & Henry, 2018). It is likely, based on existing literature on younger female victims and ISH harassment, that adult women may suffer several possible psychological impacts associated with being a victim of CSH such as symptoms of anxiety, depression, and trauma.

The experience of CSH may also be influenced by other factors. Younger women are likely to spend more time online and on social media sites and tend to have a greater number of social media followers (Zia & Amber, 2019). The amount of time spent online has also been positively correlated with cybervictimization experiences (Cross et al., 2015). Therefore, younger women may encounter more acts of CSH than older women (Selkie et al., 2015). Relationship status may influence the likelihood of individuals being exposed to acts of CSH (Dir et al., 2013). Individuals not in relationships, or in newly formed relationships, may be more likely to use online platforms for romantic pursuits, increasing the likelihood of experiencing sexually aggressive online communications (Powell & Henry, 2019). Additionally, being in a relationship has important implications for sexual harassment (Cortina & Wasti, 2005). Relationship status may serve as a proxy for perceived availability, level of protection, and vulnerability to sexual harassment (Koss et al., 1994). Women who are single or divorced may be more likely to be sexually harassed than those who are married (e.g., Lee et al., 2004). However, married women are more likely to label their experiences as sexual harassment (Olapegba, 2004). One explanation for this stems from the approach-avoidance framework which indicates that single women are more likely to interpret sexual behavior as having the potential to lead to a more positive outcome (Pina & Gannon, 2012). However, individuals in committed relationships are more likely to be inattentive to alternative relationship partners perhaps because their relationship goals are already being met (Maner et al., 2009), and may perceive uninvited sexual attention as potentially threatening to their committed relationship and to their reputation (Miller et al., 2012). Consequently, they may work to guard against such experiences to maintain their marital relationship (e.g., Petit & Ford, 2015). Lastly, due to the way CSH has been conceptualized as being separate from ISH, there is a lack of research exploring how the two relate (Ehman & Gross, 2019). It is also unknown whether individuals who experience ISH are more likely to experience CSH.

Most ISH/CSH research has focused on a relatively homogenous sample in relation to ethnicity and sexual orientation. Samples have largely consisted of white women who have identified as heterosexual. Although some research has sought to explore whether race influences the experience of CSH, the findings are largely inconclusive (e.g., Espelage et al., 2016). However, women from a minority ethnic group may report different rates of victimization following experiences of CSH. This may be because of the intersectional nature of the experiences of women from minority ethnic groups. Being both a woman and from a marginalized group may inform how they experience and make sense of being subjected to CSH (Lewis et al., 2017).

The current study explores the possible psychological impact CSH victimization may have on an under-researched sample, adult women. Further, the study aims to whether certain factors (e.g., relationship status, frequency of online behavior, and experience of ISH) relate to the experience of CSH. Two specific elements of CSH were explored: sexual coercion and unwanted sexual attention. These were chosen as they primarily focus on behaviors aimed at facilitating sexual cooperation, whereas gender harassment often refers to behaviors aimed at exerting power over women (Beck et al., 1988).

## Method

### Participants

Two hundred and seventeen adult females participated (*M* = 28.7 years, *SD* = 6.84, *n* = 172, min = 18, max = 52). A sample of 108 cases was the minimum needed to generate statistical power > 0.8 (with an alpha level of 0.05 and estimating that independent variables would account for 10% of the dependent variables) (Shieh & Kung, 2007). Thirty three percent (*n* = 46) lived with a partner/spouse, 29% (*n* = 45) were in a relationship but not cohabiting, 26% (*n* = 39) were single, and 15% (*n* = 23) were married (*N* = 153). Most participants were from a White British ethnic background (72%, *n* = 123), 14% (*n* = 8) were Black, 7% (*n* = 7) Asian and White European respectively, 7% (*n* = 4) from a mixed descent, and 4% (*n* = 2) were Arab/North African. Nearly half were educated to post-graduate level (46%, *n* = 78), 37% (*n* = 63) to undergraduate level, 11% (*n* = 19) held A levels or vocational qualifications (UK post 18 qualifications), 5% (*n* = 8) held General Certificate of Secondary Education (GCSE) qualifications (UK post 15 qualifications), and 1% (*n* = 2) held no educational qualifications. Over half (55%, *n* = 94) checked social media daily, 44% (*n* = 74) hourly; one participant did not use social media. For those did, 28% (*n* = 47) had 0 to 200 followers, 38% (*n* = 64) had 201 to 500, 16% (*n* = 26) had 501 to 1000 and 18% (*n* = 31) had more than 1,001 followers.

### Measures

*CSH.* The Cyber Sexual Experiences Questionnaire (CSEQ) (Schenk, 2008) measured experiences of CSH and contains 14 questions pertaining to unwanted sexual attention and sexual coercion and was adapted from the Sexual Experiences questionnaire (Fitzgerald et al.,1995). This questionnaire assesses unwanted requests to talk about sex online, for sexual information, and for sexual acts and assesses coercion to send sexual images and perform acts, items often missing from CSH measurements (Reed et al., 2020). Participants were asked whether they had experienced an act of CSH never, once, or more than once (coded as 0, 1, and 2). CSEQ has been used in previous research but has not been validated (Schenk, 2008). Thus, the questionnaire was validated by the current authors in an undergraduate female sample prior to its inclusion in this study (172 participants aged between 18 and 65 years) and analyzed using Confirmatory Factor Analysis. The measure was able to discriminate between the unwanted sexual attention and sexual coercion. The two-factor model was a moderate to good fit to the data [normed χ^2^(χ^2^/*df*) = 2.03, Comparative Fit Index (CFI) = 9.4, Root Mean Square Error of Approximation (RMSEA) = .08), RMSEA = 0.056, CFI = 0.921, Tucker Lewis Index (TLI)  = 0.908, Standardised Root Mean Square Residual (SRMR)  = 0.063 and χ^2^/df = 1.66] and therefore, the CSEQ was a valid questionnaire to measure CSH. The internal consistency with the study sample was α = .88. *ISH.* The original Sexual Experiences Questionnaire (Fitzgerald et al., 1995) was used to measure the ISH experiences of the sample. The questionnaire comprised of 20 items (responses coded as CSEQ) and showed good internal consistency (α = .91) within the study sample. *Anxiety and depression.* The Hospital Anxiety and Depression scale measures anxiety depression and has been widely validated (Zigmond & Snaith, 1983) and showed good internal consistency (α = .80). *Trauma.* Trauma was measured using the Post-Traumatic Stress Disorder Checklist (Weathers et al., 1993) and showed good consistency (α = .94). The participants were instructed to rank their endorsement of posttraumatic symptoms based on a five-point Likert scale (1 = not at all and 5 = extremely); overall scores were averaged. *Body image*: The Body Esteem scale (BES) (Franzoi & Shields, 1984) measured body image satisfaction across 35 items and is a multidimensional measure of body esteem used in adult populations. The BES is also gender specific; dimensions for women are sexual attractiveness, weight concern, and physical condition. Participants’ satisfaction with various body parts and functions is rated on a five-point Likert scale (1 = strong negative feeling, 5 = strong positive feelings). The scale showed good internal consistency (α = .93); scores were averaged to create a composite score. *General online victimization*. A sub-scale on the General Online Victimization questionnaire (Tynes et al., 2010) pertaining to participants’ experiences of general online victimization was used and showed good internal consistency within the current sample (α = .78). The scores on the online victimization scale were based on a six-point Likert scale (0 = never, 5 = everyday).

### Procedure

Ethical approval was given by the researchers’ University’s Central Ethics Committee who adhered to the British Psychological Society’s (BPS) Code of Ethics and Conduct (BPS, 2021). Participants were treated with integrity, honesty, and openness to ensure power balance in the research (e.g., they were fully informed, provided informed consent, thanked for time, remained anonymous, and their responses were stored confidentially). Participants completed the study online; questionnaires were presented in a random order, and participants completed demographic details such as their age, relationship status, and whether they had experienced CSH at the end of the survey. The survey took approximately 20 min to complete. Participants were fully debriefed and given contact detail to services for victim support, rape and sexual abuse, as well as the police.

### Design and Analysis

The study adopted a cross-sectional, quasi-experimental design. To explore psychological impact of CSH, appropriate inferential statistics were adopted to compare average scores on the relevant scales between participants who had experienced CSH and those who had not. To examine the relationship between demographic and social factors, experience of CSH and psychological impact, correlations and multiple linear regressions were adopted.

## Results

### Psychological Impact of CSH

Average scores were as follows: CSH (*M* = 1.49, *SD* = 0.54), ISH experiences (*M* = 1.65, *SD* = 0.47), anxiety/depression (*M* = 2.16, *SD* = 0.44), trauma (*M* = 2.33, *SD* = 0.90) and body image dissatisfaction (*M* = 3.08, *SD* = 0.65). Forty-four percent of participants (*n* = 74) reported experience of CSH within their lifetime, while 56% (*n* = 94) did not. Participants who experienced CSH had significantly higher depression/anxiety scores (*M* = 2.27, *SD* = 0.45) than those who had not (*M* = 2.06, *SD* = 0.39) (*t*(166) = 3.15, *p* < .05) and also reported significantly higher trauma (*M* = 2.58, *SD* = 0.88) than those who did not (*M* = 2.14, *SD* = 0.83), *t*(205) = 3.31, *p* < .05. Levene’s test for equality of variances was significant for body image and ISH and so an adjustment to the degrees of freedom was conducted (Welch-Satterthwaite method; Pallant, 2010). Those who reported CSH experienced higher levels of ISH (*M* = 1.86, *SD* = 0.50) than those who did not (*M* = 1.48, *SD* = 0.35); *t*(127) = 5.56, *p* < .001) and reported lower levels of body image satisfaction (*M* = 2.93, *SD* = 0.53) in comparison to those who did not (*M* = 3.21, *SD* = 0.69), *t*(166) = −2.98, *p* = .003).

### Relationship Between Demographic and Social Factors, Experience of CSH and Psychological Impact

The Pearson’s correlation matrix (Table 1) showed CSH was positively correlated with ISH, anxiety/depression and trauma symptoms, number of social media followers, and the frequency of checking social media and negatively correlated with body image satisfaction, age, the length of time a person was in a relationship and a person’s highest level of educational attainment. These associations needed to be statistically controlled in the regression analysis and so were added to step 1.

**Table 1.** Pearson’s Correlation for Demographic Variables and Psychological Variables Associated with CSH.

Variables	1	2	3	4	5	6	7	8	9	10	11
1 Age	—										
2 Relationship length (months)	.40***	—									
3 Frequency checking social media	−.06	−.02	—								
4 Number social media followers	−.25**	−.14	−.04	—							
5 Highest level education	.14	.05	−.15	−.01	—						
6 Online cybervictimization	−.16*	−.21*	.05	.22**	−.11	—					
7 Anxiety/depression	−.20**	−.11	.12	.10	−.23**	.30***	—				
8 Body image	−.10	−.14	−.98	−.020	.15	−.21**	−.43***	—			
9 Trauma	−.25**	−.28*	.17*	.09	−.29***	.37***	.75***	−.44***	—		
10 ISH	−.20**	−.35***	.12	.18*	−.22**	.31***	.31***	−.27***	.42***	—	
11 CSH	−.16*	−.32***	.28**	.22**	−.37***	.38***	.36***	−.23**	.41***	.69***	—

*Note.* CSH = cyber sexual harassment; ISH = in-person sexual harassment.

* *p* < .05. **p < .01. ***p < .001.

Three separate hierarchical multiple linear regressions examined the association between trauma, body image, depression/anxiety, and CSH while controlling for each relevant variable outlined above separately. In step 1 of each model, age, the length of the participant’s current relationship, their number of social media followers, their frequency of social media use and their level of general online victimization were entered. Number of social media followers, level of general online victimization, and the frequency of social media use were entered as categorical variables, while age and the length of relationship were entered as continuous variables. CSH was then entered into step 2, to examine the incremental variance CSH added to predicting trauma, body image, and depression/anxiety. ISH was entered into step 3 to see if CSH would continue to add unique variance (order variables were entered was based on existing guidance; Pallant, 2010). Regression analysis then compared the models generated from each of the steps to determine if CSH predicted each psychological variable over and above demographic variables. If the model with ISH was significant, this would indicate ISH was an important factor to explaining variance in the dependent variables and above the effect of CSH. Thus, this would suggest ISH (which is correlated with CSH) should be prioritized in mental health interventions along with CSH. Additionally, if CSH’s unique predictive power was reduced upon entering offline harassment, then this would indicate offline harassment took significant variance away from CSH.

#### Anxiety and Depression

Covariates explained 18.4% of the variance in anxiety and depression (see Table 2). The addition of CSH in step 2 accounted for a significant increase of 4% in the variance explaining anxiety/depression, ∆*R*^2^ = .04, *F*(1, 128) = 6.31, *p* = .01. When ISH was added, it was not significant, ∆*R*^2^ = .002, *F*(1, 127) = 0.38, *p* = .54. Therefore, CSH was a significant predictor, and ISH did not add to our understanding of anxiety/depression over and above CSH. CSH’s beta dropped to non-significance in the final model.

**Table 2.** Summary of Hierarchical Multiple Regression Model Predicting Anxiety/Depression.

Predictor Variables	β	*SE B*	Lower Confidence Interval	Upper Confidence Interval	β	*p*
Step 1						
Intercept	2.59	0.24	2.12	3.07		< .001***
Social media followers	−.02	0.03	−0.08	0.05	−.04	.62
Education level	−.09	0.03	−0.16	−0.02	−.22	.01**
Length of current relationship	9e−5	6.6e−4	−0.001	0.001	.01	.89
Age	−.01	0.01	−0.02	6.6e−4	−.17	.06
How often you check social media	.09	0.06	−0.04	0.20	.11	.17
General online victimization	.17	0.06	0.06	0.28	.26	.003**
Step 2						
Intercept	2.16	0.29	1.58	2.73		< .001***
Social media followers	−.04	0.04	−0.11	0.03	−.11	.22
Education level	−.05	0.04	−0.12	0.02	−.12	.18
Length of current relationship	5.75e−4	6.78e−4	−7.66e−4	0.002	.08	.40
Age	−.01	0.01	−0.02	0.001	−.16	.08
Frequency checking social media	.06	0.06	−0.06	0.18	.08	.32
General online victimization	.12	0.06	−0.002	0.23	.17	.05
CSH	.24	0.09	0.05	0.42	.27	.01*
Step 3						
Intercept	2.12	0.30	1.52	2.71		< .001***
Social media followers	−.04	0.03	−0.11	0.03	−.11	.22
Education level	−.05	0.04	−0.12	0.02	−.12	.17
Length of current relationship	6.29e−4	6.85e−4	−7.270e−4	0.002	.08	.36
Age	−.01	0.01	−0.02	0.001	−.16	.08
Frequency checking social media	.05	0.06	−0.07	0.17	.07	.40
General online victimization	.11	0.06	−0.003	0.23	.17	.06
CSH	.20	0.11	−0.01	0.42	.23	.07
ISH	.06	0.10	−0.14	0.27	.07	.54

*Note.* Step 1: *R*^2^ = .184, *F*(6, 127) = 4.84, *p* < .001; Step 2: *R*^2^ = .222, *F*(7, 128) = 5.22, *p* < .001; Step 3: *R*^2^ = .225, *F*(8, 129) = 4.60, *p* < .001. CSH = cyber sexual harassment; ISH = in-person sexual harassment.

* *p* < .05. **p < .01. ***p < .001.

#### Body Image

Covariates explained 12.8% of the variance in body image (Table 3). The addition of CSH in step two accounted for an increase of 3.8% in the variance explaining body image. The change in *R*^2^ was significant, ∆*R*^2^ = .04, *F*(1, 128) = 5.81, *p* = .02. When ISH was included, it became a significant predictor of body image, ∆*R*^2^ = .03, *F*(1, 127) = 4.82, *p* = .03. However, at step 3, CSH was no longer a statistically significant predictor of body-image dissatisfaction; ISH is more important than CSH in explaining body image dissatisfaction.

**Table 3.** Summary of Hierarchical Multiple Regression Model Predicting for Body Image.

Predictor Variables	β	*SE B*	Lower Confidence Interval	Upper Confidence Interval	β	*p*
Step 1						
Intercept	3.18	0.35	2.50	3.87		< .001
Social media followers	.02	0.05	−0.08	0.12	.03	.71
Education level	.12	0.05	0.02	0.21	.20	.02
Length of current relationship	−.002	9.63e−4	−0.004	1.31e−4	−.17	.07
Age	−.004	0.01	−0.02	0.01	−.05	.62
How often you check social media	−.09	0.09	−0.27	0.08	−.09	.27
General online victimization	−.23	0.08	−0.39	−0.68	−.24	.01**
Step 2						
Intercept	3.78	0.43	2.94	4.62		< .001***
Social media followers	.05	0.05	−0.05	0.16	.09	.29
Education level	.06	0.05	−0.04	0.17	.10	.26
Length of current relationship	−.002	9.88e−4	−0.004	−4.87e−4	−.23	.02
Age	−.01	0.01	−0.02	0.01	−.06	.53
How often you check social media	−.06	0.01	−0.24	0.11	−.06	.46
General online victimization	−.15	0.09	−0.32	0.02	−.16	.08
CSH	−.32	0.14	−0.59	−0.05	−.27	.02*
Step 3						
Intercept	3.99	0.43	3.14	4.84		< .001***
Social media followers	.06	0.05	−0.04	0.16	.10	.25
Education level	.07	0.05	−0.04	0.17	.11	.21
Length of current relationship	−.003	9.8e−4	−0.01	−7.77e−4	−.26	.01*
Age	−.01	0.01	−0.02	0.01	−.06	.52
Frequency checking social media	−.02	0.09	−0.20	0.15	−.02	.81
General online victimization	−.15	0.09	−0.31	0.02	−.16	.09
CSH	−.15	0.16	−0.45	0.16	−.12	.35
ISH	−.33	0.15	−0.62	−0.04	−.25	.03*

*Note.* Step 1: *R*^2^ = .128, *F*(6, 129) = 3.15, *p* < .01; Step 2: *R*^2^ = .166, *F*(7, 128) = 3.63, *p* < .001; Step 3: *R*^2^ = .196, *F*(8, 127) = 3.88, *p* < .001. CSH = cyber sexual harassment; ISH = in-person sexual harassment.

* *p* < .05. **p < .01. ***p < .001.

#### Trauma

The step including the covariates explained 32.2% of the variance in trauma (see Table 4). Adding CSH in step two resulted in a 3.4% increase in variance, ∆*R*^2^ = .034, *F*(1, 128) = 6.69, *p* = .011. In step three, when ISH was included, CSH ceased to be a significant predictor of trauma and change in *R*^2^ was not statistically significant, ∆*R*^2^ = .015, *F*(1, 127) = 3.10, *p* = .081; contribution of ISH did not significantly account for additional variance in trauma. In the final model, CSH ceased to be a significant predictor of trauma. Thus, CSH adds significant variance to the understanding of trauma when including covariates, but in competition with ISH, it was not significant, possibly due to covariance.

**Table 4.** Summary of Hierarchical Multiple Regression Model Predicting Trauma.

Predictor Variables	β	*SE B*	Lower Confidence Interval	Upper Confidence Interval	β	*p*
Step 1						
Intercept	3.58	0.42	2.74	4.41		< .001***
Social media followers	−.03	0.06	−0.14	0.09	−.03	.66
Education level	−.22	0.06	−0.34	−0.11	−.28	< .001***
Length of current relationship	−9.81e−4	0.001	−0.003	0.001	−.07	.40
Age	−.03	0.01	−0.05	−0.01	−.24	.004**
Frequency checking social media	.22	0.11	0.01	0.43	.15	.04
General online victimization	.37	0.09	0.17	0.56	.29	< .001***
Step 2						
Intercept	2.79	0.51	1.77	3.80		< .001
Social media followers	−.07	0.06	−0.19	0.05	−.09	.23
Education level	−.15	0.06	−0.28	−0.03	−.19	.02*
Length of current relationship	−1.02e−4	0.001	0.002	0.002	−.01	.93
Age	−.03	0.01	−0.05	−0.01	−.23	.01**
Frequency checking social media	.18	0.11	−0.03	0.39	.12	.09
General online victimization	.27	0.10	0.06	0.47	.21	.01*
CSH	.43	0.17	0.10	0.75	.26	.01*
Step 3						
Intercept	2.59	0.52	1.55	3.62		< .001***
Social media followers	−.08	0.06	−0.20	0.04	−.01	.21
Education level	−.16	0.06	−0.28	−0.03	−.20	.02*
Length of current relationship	1.62e−4	0.001	−0.002	0.003	.01	.90
Age	−.03	0.01	−0.05	−0.01	−.23	.01**
Frequency checking social media	.14	0.11	−0.07	0.35	.09	.207
General online victimization	.26	0.10	0.06	0.46	.20	.01*
CSH	.26	0.19	−0.12	0.63	.16	.18
ISH	.31	0.18	−0.04	0.67	.17	.08

*Note.* Step 1: *R*^2^ = .322, *F* (6, 129) = 10.2, *p* < .001; Step 2: *R*^2^ = .355, *F* (7, 128) = 10.08, *p* < .001; Step 3: *R*^2^ = .371, *F* (8, 127) = 9.35, *p* < .001. CSH = cyber sexual harassment; ISH = in-person sexual harassment.

* *p* < .05. **p < .01. ***p < .001.

## Discussion

At present, very little is known about the psychological impact of CSH on adult women. Study findings were consistent with existing research on ISH (e.g., Bendixen et al., 2018; Fisher et al., 2019; Marsh et al., 2009; Richman et al., 1999) and CSH victimization within adolescent and university samples (Cripps & Stermac, 2018; Stahl & Dennhag, 2021); women experiencing CSH reported higher levels of trauma, anxiety, and body image dissatisfaction than those who did not, even when controlling for demographic and social variables. CSH therefore victimization impacts well-being and self-esteem within adult women and can compound pre-formed negative beliefs of body image (Fredrickson & Roberts, 1997; Lewis-Smith et al., 2019). Interestingly, ISH experience was a significant predictor of body image dissatisfaction over and above CSH. We know that ISH can impact upon perceptions of one’s own body, leading women to have a heightened awareness and to place higher value on their external appearance (Lindberg et al., 2007), which also seems to happen in CSH. Further research is needed to explore why ISH impacts body image satisfaction over and above CSH; although the experience of CSH and ISH are highly correlated, the way in which they impact perceptions of a person’s own body may be different.

Social and demographic factors were also related to cyber-victimization; women experiencing CSH were more likely to have experienced ISH, perhaps reflecting the pervasive and multi-faceted nature of CSH. However, there was little evidence to suggest CSH predicts trauma, depression, anxiety, or body esteem over and above ISH. Future research should examine the relative impact of each type of harassment to see the extent to which this may vary, quantitatively and qualitatively.

Younger participants, those who more frequently checked their social media accounts, and those who had a greater number of social media followers were found to have more experience with CSH. This is not surprising considering the more time spent online has been related to more general cybervictimization experiences (Cross et al., 2015), and that younger women tend to spend more time online and have more social media followers (Zia & Amber, 2019). Women with larger social media followings are also often subjected to greater levels of objectification and sexually aggressive comments (Drenten et al., 2019). General experiences of online victimization were significantly associated with trauma in step three of the model in addition to age, with younger participants also reporting higher levels of trauma. Non-sexual negative online experiences can be experienced as traumatic in younger populations perhaps due to their greater usage of social media platforms, the importance of online social networks, and the frequency by which they are exposed to negative online comments (Ybarra & Mitchell, 2004).

Lastly, the length of time a participant had been in a relationship was negatively correlated with experience of CSH; the less time participants were in a relationship, the more likely they were to experience CSH. This is analogous to previous research; women who are single are more likely to be sexually harassed than those in a relationship (Lee et al., 2004).

### Limitations

The cross-sectional nature of the study means causality could not be determined. Future research should also examine how CSH could impact psychological well-being over time. Further, as the study relied on a self-report method, there is the possibility of recall bias; individuals experiencing acts of CSH who are anxious/depressed recall these instances differently in comparison to a person who is not experiencing the same psychological difficulties (Sanz, 1996). Additionally, the questionnaire did not ask participants questions related to the severity of the CSH. This omission makes it difficult to ascertain the fear generated by specific experiences of CSH and more challenging to identify whether there are specific experiences are more strongly correlated with the psychological distress associated with CSH (Drebing et al., 2014). There is a need to measure further aspects of CSH in studies such as context (whether the CSH occurs in private versus group messages), whether the perpetrator sends a personal sexual image (e.g., “dick pic”), and whether the victim had sexual images/messages shared without consent (Reed et al., 2020).

The sample of participants was largely white and heterosexual, similar to previous studies (Moradi, 2017). Individuals from LGBT communities and minority ethnic groups may be more likely to experience online victimization (Lenhart et al., 2016). Although some research has sought to explore whether race influences the experience of CSH, the findings are largely inconclusive (e.g., Espelage et al., 2016). Women from a minority ethnic group may report different rates of victimization following experiences of CSH, due to the intersectional nature of the experiences of these women. Furthermore, women who embody a marginalized identity may experience a wide variety of cyber-negative acts reflecting their overlapping identities (Lenhart et al., 2016). The generalizability of the study findings would be improved if it contained a more diverse sample.

### Clinical Implications and Future Research

As the world becomes increasingly digital, many interactions will take place over an online format or will supplement in-person contacts. While CSH ceased to be a significant predictor when ISH was entered into the model, individuals experiencing CSH tended to report higher levels of trauma, body image dissatisfaction and anxiety/depression. The findings highlighted the negative impact CSH can have, particularly on young women and individuals experiencing other forms of harassment. As social media is becoming increasingly important in how people connect with others, technological communication may have a significant impact on the quality of people’s relationships (Deady et al., 2017). Therefore, individuals accessing mental health services should be routinely asked about their online interactions and whether this is a source of distress for them. In situations where individuals report having difficulties with their body image, clinicians may want to specifically ask individuals about more general experiences of cybervictimization (e.g., when online, do people say mean things about the way you look?). Clinicians may also want to ask participants about the different social media sites clients used and the type of material they see while online. This can help to identify whether clients are being exposed to online materials increasing their level of distress or contributes to negative perceptions they may have of themselves (e.g., body dissatisfaction). Through asking individuals about their online use, the negative impact which online interactions may have could be explored and validated in a safe setting.

CSH research is still in its infancy, and future research should seek to examine the persistence, consistency, and emotional impact of this type of victimization. This will help to improve the specificity of interventions when working with those experiencing cybervictimization. More specifically, future research should conduct longitudinal studies examining different types of victimizations (online and in-person) and the short and long-term effects of their experiences, as well as the platforms through which they are victimized, as previous research has shown medium cybervictimization is experienced through, impacts on the severity of the distress experienced (Peled, 2019). Researchers could also explore whether the intrusiveness of specific technological mediums affect psychological well-being. Lastly, there is a need to consider whether characteristics of the offense (e.g., language used and severity) and post-event characteristics (e.g., victim’s coping strategies and social support) could influence the extent to which adverse effects are experienced (O’Brien & Burrell, 2020).

## Conclusion

CSH can disrupt the normal interactions people have in their everyday lives. The trauma and shame accompanying severe CSH experiences may cause women to avoid digital spaces. This can potentially limit the opportunity for women to develop meaningful relationships and can also inhibit their financial opportunities. The present study, therefore, highlights the need to understand the nuances of CSH, making it a unique form of victimization. This will enable a contextual understanding of how different forms of cybervictimization impact on the individual.
